# Understanding the Role of M13 Bacteriophage Thin Films on a Metallic Nanostructure through a Standard and Dynamic Model

**DOI:** 10.3390/s23136011

**Published:** 2023-06-28

**Authors:** Thanh Mien Nguyen, Cheol Woong Choi, Ji-Eun Lee, Damun Heo, Ye-Won Lee, Sun-Hwa Gu, Eun Jeong Choi, Jong-Min Lee, Vasanthan Devaraj, Jin-Woo Oh

**Affiliations:** 1 Bio-IT Fusion Technology Research Institute, Pusan National University, Busan 46241, Republic of Korea; 2 Department of Internal Medicine, Medical Research Institute and Research Institute for Convergence of Biomedical Science and Technology, Pusan National University Yangsan Hospital, Yangsan-si 50612, Republic of Korea; 3 School of Medicine, Pusan National University, Yangsan 50612, Republic of Korea; 4 Department of Ophthalmology, Research Institute for Convergence of Biomedical Science and Technology, Pusan National University Yangsan Hospital, Yangsan 50612, Republic of Korea; 5 School of Nano Convergence Technology, Hallym University, Chuncheon 24252, Republic of Koreayw330@naver.com (Y.-W.L.); 20203301@hallym.ac.kr (S.-H.G.); 6 Center of Nano Convergence Technology, Hallym University, Chuncheon 24252, Republic of Korea; 7 Department of Nanoenergy Engineering and Research Center for Energy Convergence Technology, Pusan National University, Busan 46214, Republic of Korea

**Keywords:** M13 bacteriophage, metallic nanoparticles, plasmonics, dynamic response, self-assembly, simulations

## Abstract

The dynamic and surface manipulation of the M13 bacteriophage via the meeting application demands the creation of a pathway to design efficient applications with high selectivity and responsivity rates. Here, we report the role of the M13 bacteriophage thin film layer that is deposited on an optical nanostructure involving gold nanoparticles/SiO_2_/Si, as well as its influence on optical and geometrical properties. The thickness of the M13 bacteriophage layer was controlled by varying either the concentration or humidity exposure levels, and optical studies were conducted. We designed a standard and dynamic model based upon three-dimensional finite-difference time–domain (3D FDTD) simulations that distinguished the respective necessity of each model under variable conditions. As seen in the experiments, the origin of respective peak wavelength positions was addressed in detail with the help of simulations. The importance of the dynamic model was noted when humidity-based experiments were conducted. Upon introducing varied humidity levels, the dynamic model predicted changes in plasmonic properties as a function of changes in NP positioning, gap size, and effective index (this approach agreed with the experiments and simulated results). We believe that this work will provide fundamental insight into understanding and interpreting the geometrical and optical properties of the nanostructures that involve the M13 bacteriophage. By combining such significant plasmonic properties with the numerous benefits of M13 bacteriophage (like low-cost fabrication, multi-wavelength optical characteristics devised from a single structure, reproducibility, reversible characteristics, and surface modification to suit application requirements), it is possible to develop highly efficient integrated plasmonic biomaterial-based sensor nanostructures.

## 1. Introduction

The properties of biomaterials have opened pathways in various multi-disciplinary applications, like bio-optics, regenerative medicine, tissue engineering, energy devices, sensors, and point-of-care devices [1,2,3,4,5,6]. Notably, in the past decade, M13 bacteriophage (hereafter termed M13 bacteriophage) attracted significant interest with regard to a wide variety of applications owing to its superior self-assembly properties and manipulation of surface coat proteins relevant to application requirements [7,8,9]. The M13 bacteriophage that involved self-assembled nanostructures revealed a repeatable and regular formation of densely packed building blocks, a pattern that is highly beneficial for designing and developing efficient devices per unit volume [7,8]. This strategy allows us to build and control the basic structural building blocks in a highly ordered and systematic manner through self-assembly techniques (which were inspired by biological systems or the biomimetic approach) [7,10,11,12,13,14]. Furthermore, in the synthesis approach, handling properties of the M13 bacteriophage were easier and non-toxic, which is essential in employing these biomaterials as templates. In addition to the above benefits, these highly ordered nanostructures can be fabricated on a large scale using a complex free, straightforward self-assembly method at a relatively low-cost [15,16,17,18,19,20].

Geometrically, the M13 bacteriophage is nanofibrous with a diameter of ~6.6 nm and a height of ~880 nm [21,22,23,24,25]. The bottom and top ends of the M13 bacteriophage consist of five to seven copies of the pVII, pIX, pVI, and pIII coat proteins. Moreover, 2700 helically arranged copies of the pVIII major coat protein are located on its body’s surface. With the help of the genetic engineering process, it is possible to engineer the major coat protein according to the application requirements. The advantages of utilizing surface coat protein engineering are that suiting application demands, environmentally friendly synthesis, and design of highly ordered reproducible nanostructures on a low-cost scale open various exciting applications in the fields of photocatalysis [18,26,27], optical nanostructures [19,21,22,28], point-of-care diagnostics [13,29], environmental care [30,31], sensors [18,19,21], solar device [32,33], nanogenerators [10,20], etc.

In particular, as the role of M13 bacteriophage in the sensor field has experienced enormously growth in great demand, it is necessary to understand how the M13 bacteriophage interacts with and influences the optical and geometrical properties when it is integrated with a plasmonic or photonic nanostructure [34,35,36,37,38,39,40,41,42]. The availability of large field local enhancement (bright optical mode) devised from these optical nanostructures, alongside the M13 bacteriophage’s numerous benefits, could significantly engineer a variety of areas involving non-linear optics, plasmonic/photonic sensors, SERS, and biomedical applications.

In this study, we conduct an in-depth examination of the optical and structural properties of M13 bacteriophage/gold nanoparticles/SiO_2_/Si nanostructures. The nano-gap effect in plasmonics could act as an attractive candidate in testing the M13 bacteriophage’s capabilities [43,44]. Previous research often incorporated the M13 bacteriophage as part of the plasmonic nanogap, which consequently restricted its applications. Given the biomaterial nature of M13 bacteriophage, there are limitations to the types of fabrication methods that can be used, especially those not involving high temperatures or exposure to high energy. However, our proposed structures were fabricated through an additional spin-coating step after the creation of the plasmonic nanostructures. This methodology grants us substantial flexibility in choosing a fabrication process for the plasmonic nanostructures, allowing us to utilize any form currently in use. Recognizing that the M13 bacteriophage can undergo structural and optical variations in response to external stimuli, we exposed it to varying levels of humidity to explore its multi-wavelength optical properties within a single sample study. We observed that as humidity increased from 20 to 90%, the reflectance peak of this structure shifted by approximately 70 nm. Three-dimensional FDTD simulations were performed to characterize the optical properties.

## 2. Materials and Methods

### 2.1. Fabrication of Plasmonic Nanoparticle—M13 Bacteriophage Biomaterial Thin Film Nanostructure

**Figure 1:**
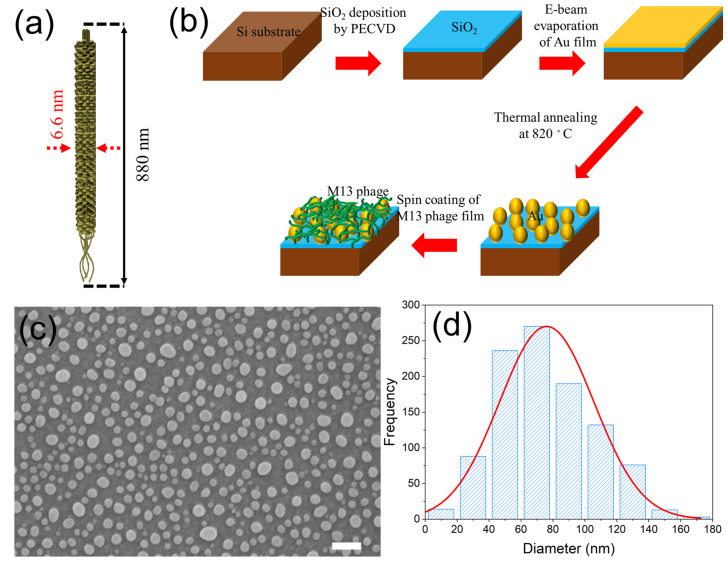
(**a**) A schematic diagram of M13 bacteriophage that shows that is a nanowire that is 6.6 nm wide and 880 nm long. (**b**) A fabrication scheme of plasmonic nanoparticle–M13 bacteriophage biomaterial thin film nanostructure on a SiO_2_/Si substrate. (**c**) A SEM image of fabricated Au NPs on the SiO_2_/Si. The scale bar is 200 nm. (**d**) The size distribution of Au NPs that display a mean size of ~73.6 nm. The red line is the result of the Gaussian fitting.

The schematic of the fabrication process is shown in Figure 1. We prepared a Si wafer, which went through a cleaning process in the following order: acetone, methanol, and isopropyl alcohol. A 300 nm thick SiO_2_ film was deposited onto a Si wafer via the PECVD (plasma-enhanced chemical vapor deposition) method at 350 °C. Gold film with a thickness of 9 nm was deposited onto the SiO_2_/Si via an electron beam evaporation. The deposition rate of gold was maintained at 0.06 nm s^−1^ at room temperature. The fabricated substrate was sliced into 2 cm × 2 cm pieces and loaded into a thermal annealing chamber, using a halogen lamp as a heating source. The thermal annealing chamber was pumped down to vacuum for 2 h, and a chamber pressure of 1.8 mTorr was created by introducing nitrogen gas. The gold-coated substrate was then annealed at 820 °C for 2 min to form the gold nanoparticles. Before coating M13 the bacteriophage, the cleaning process Ag NPs/SiO_2_/Si was repeated and dried with nitrogen gas. M13 bacteriophage suspension was prepared by dispersion in Tris-buffered saline buffer (37.5 mM NaCl and 12.5 mM of Tris, with a pH condition of 7.5). The nanostructured biomaterial thin film on Au NPs/SiO_2_/Si substrate was fabricated via spin coating of M13 bacteriophage (1500 rpm, 2 min). The M13 bacteriophage film’s thickness (t) was controlled by varying concentrations (6 mg/mL, 8 mg/mL, and 10 mg/mL). The M13 bacteriophage suspension’s concentration was confirmed via a UV-Visible spectrometer (EVO300PC, Thermo Scientific, Waltham, MA, USA) and Beer–Lambert law calculation [7,34].

### 2.2. Atomic Force Microscopy

We examined the surface topography of samples utilized in this work using an NX10 AFM system in non-contact mode (NX10, Park Systems, Suwon, Republic of Korea). We utilized a specialized probe (PPP-NCHR, NANOSENSORS, Neuchatel, Switzerland) for non-contact mode. The XY directional scanning of AFM was operated via a XEP 3.0.4 data acquisition program and post-processed via XEI 1.8.2 image processing software (Park Systems, Republic of Korea).

### 2.3. Optical Measurements

The reflectance spectra of the plasmonic NP–biomaterial thin film nanostructure were measured using a commercial Olympus bright-field/dark-field (BF/DF) microscope (BX53M) with a 100X 0.9 NA objective. The color film was illuminated using an unpolarized halogen light. The reflectance of the M13 bacteriophage color film was characterized using a fiber-optic spectrometer (US/USB4000, Ocean Optics, Dunedin, FL, USA) under a reference to the white reflectance standard. Dark-field images were captured via a dark-field (DF) microscope (BX53M, Olympus, Tokyo, Japan). An unpolarized halogen lamp was used to illuminate the sample(s), and the collection of scattered light from the sample was performed using the objective lens (100X, 0.9 NA, respectively) in a DF mode. The color images were recorded in BF/DF mode with the help of a 16 MP CMOS camera with a sensor size of 1/2.33” and a built-in integrated image–signal processor (DigiRetina16, Tucsen Photonics, Fuzhou, China) connected to the Olympus microscope. All measurements were carried out in a dark room to prevent external light interference. With the help of our home-built chamber system, we were able to control the humidity levels input. At first, we removed any presence of moisture found in the home-built chamber (with the sample) with the help of dry N_2_ gas, and we confirmed this step with the humidity sensor. The input of humidity levels (moisture flow) varied from 20 to 90% in 10% steps.

### 2.4. Three-Dimensional Electromagnetic Simulations

A three-dimensional (3D) electromagnetic Maxwell solver utilizing the finite-difference time–domain method (FDTD) was employed to carry out optical simulations (ANSYS Lumerical FDTD, Vancouver, BC, Canada) [43,44]. A plane wave source was used to illuminate the plasmonic NP–thin film nanostructure (surrounded by air (n = 1) on top) with an incident electric field of E_0_ (see Supporting Information or SI Figure S1). The nanostructure was surrounded by periodic boundary conditions in the XY direction and a perfectly matched layer (PML) boundary in the Z direction. A cross-sectional power monitor was placed close to the plasmonic NP–biomaterial thin film nanostructure to capture XZ electric field amplitude profiles. A power monitor positioned above the plane wave source recorded the reflectance spectra. A general mesh size of 5 nm was applied to the entire simulated region, and a mesh override of 0.5 nm was applied in proximity to the NP region. We selected the Au NP diameter “D” as 70 nm, with the interparticle spacing, or “gap”, between NPs set at 10 nm. The chosen material’s refractive indices were as follows: Johnson and Christy database for gold, Palik database for Si and SiO_2_, and n = 1.37 were applied for M13 bacteriophage [34,45,46].

## 3. Results and Discussion

### 3.1. Fabrication Analysis and Optical Properties

**Figure 2:**
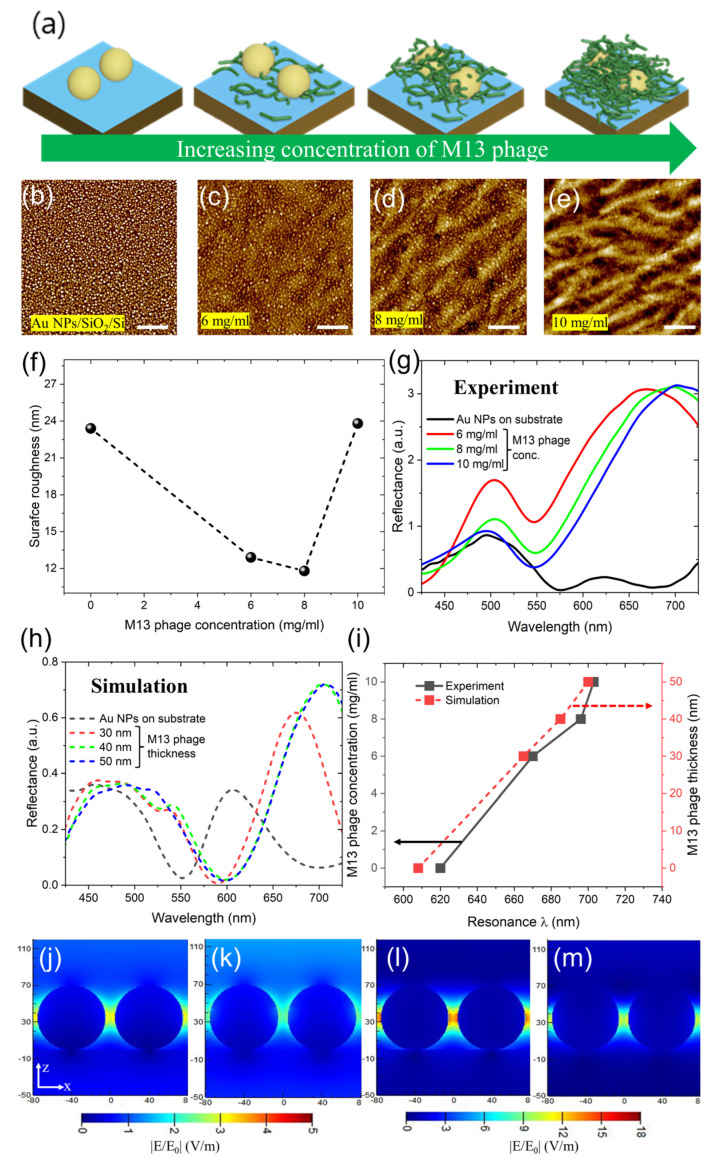
(**a**) A schematic of M13 bacteriophage deposition on top of the Au NPs as a function of increased concentration. Two-dimensional AFM scan profiles of Au NPs/SiO_2_/Si (**b**) and deposition of M13 bacteriophage biomaterial on its top as a function of 6 mg/mL (**c**), 8 mg/mL (**d**), and 10 mg/mL (**e**) concentrations. The scale bar is 2 µm. (**f**) The surface quality of the film was extracted from the AFM data as a function of the M13 bacteriophage’s concentration (0 indicates no M13 bacteriophage). Experimental (**g**) and simulated (**h**) reflectance spectra for the Au NPs on the substrate with and without M13 bacteriophage deposition. (**i**) Related to M13 bacteriophage’s concentration and thickness estimations from simulations as functions of longer wavelength λ resonance positions. Simulated cross-sectional XZ electric field amplitude profiles that display NP mode- (**j**,**k**) and gap mode (**l**,**m**)-based optical characteristics for Au NP structures and M13 bacteriophage-coated Au NP structures, respectively. The “x” and “z” axes numbers are measured in nanometers.

Figure 2a schematically describes how the concentration of M13 bacteriophage could help to adjust the biomaterial layer thickness on top of Au NPs/SiO_2_/Si. After spin coating M13 bacteriophage with variable concentrations, it began to fill the spaces between and on top of the Au NPs. This prediction was evident from AFM images, as shown in Figure 2b–e. Before M13 bacteriophage deposition, due to the height difference of Au NPs from the SiO_2_ film, it was expected to have poor surface film quality (surface roughness). As observed for 6 mg/mL and 8 mg/mL M13 bacteriophage concentrations, it tends to fill the spaces between and above NP (majorly). Due to this deposition approach, the surface roughness of the film quality improves from 23.4 (Au NPs only on a substrate) to 11.8 nm (8 mg/mL M13 bacteriophage concentration), as shown in Figure 2f. At 10 mg/mL concentration, we can observe two kinds of cases happening dominantly on Au NPs/SiO_2_/Si: firstly, filling of the spacings between the NPs is noted; and secondly, the geometrical nature of M13 bacteriophage combines to form micron-sized bundled rod structures due to its increased density and binding nature. Due to this outcome, the thin film surface quality jumps from 11.8 to 23.8 nm, which is of a similar condition as that recorded for sole use of bare NPs (Figure 2f).

We carried out reflectance measurement on these samples, and a redshift in its resonance wavelength position as a function of M13 bacteriophage thickness or concentration was noted (Figure 2g). After the deposition of M13 bacteriophage film atop the NP, the longer wavelength resonance positions shifted from 620 (Au NPs/SiO_2_/Si) to 670 nm (6 mg/mL M13 bacteriophage concentration). By increasing the M13 bacteriophage concentration or, in other words, the bacteriophage film thickness, the longer wavelength resonance peak position further shifted to 703 nm (10 mg/mL M13 bacteriophage concentration). To further validate the optical properties of resonance wavelength positions and M13 bacteriophage film thickness dependence, we carried out three-dimensional finite-difference time–domain (3D FDTD) simulations. At first, we simulated either a single NP model or a randomly positioned multiple NP model with PML boundary conditions in XYZ directions, which is considered a general approach (see SI Figure S2). As seen in SI Figure S2a, a single NP model’s (or, in other words, a randomly positioned NPs model) reflectance results do not agree with the experimental results. The dimer model involving two NPs separated by a gap distance of 10 nm also disagrees with experimental results (SI Figure S2b). We found that a periodic NP model with interparticle spacing or gap size of 10 nm (Au NPs/SiO_2_/Si) very much agrees with the experimental data, as shown in Figure 2h,i. As a base design, the simulated periodic model and experimental data very much agree with Au NPs on a substrate. Retaining this a fundamental design, we simulated the M13 bacteriophage’s thickness, with tests ranging from 30 to 50 nm in 10-nanometer steps. The reflectance spectra trend between the experiment and simulation very much agrees, thereby predicting the M13 bacteriophage’s thickness (upon different concentrations used).

The AFM line scan over the M13 bacteriophage deposited samples revealed a similar thickness profile to that applied in simulations (6 mg/mL was ~30 nm, 8 mg/mL was ~40 nm, and 10 mg/mL was ~50 nm), which seems to be reasonably applicable (SI Figure S3). Two kinds of peak resonance positions were observed from both experimental and simulated reflectance spectra. NP mode-based optical characteristics were observed at shorter wavelength resonances of ~500 nm. The longer wavelength resonance positions correspond to gap-mode-based properties (607 nm for NPs only: ~700 nm for NPs and M13 bacteriophage). We extracted cross-section XZ electric field amplitude profiles to explain these optical mode differences. The NP mode-based properties are shown in Figure 2j,k’s cross-sectional XZ electric field amplitude profiles taken at shorter wavelength resonance positions for Au NPs and M13 bacteriophage-coated Au NPs on the substrate. In the case of Figure 2l,m, gap mode-based characteristics were displayed from cross-sectional XZ electric field amplitude profiles taken at longer wavelength resonance positions for Au NPs and M13 bacteriophage-coated Au NPs on the substrate. The electric field amplitude strength differences can understand the clear distinguishability between NP and gap mode: less near-field strength for NP mode-based and higher near-field enhancement for gap mode-based resonance wavelengths peak positions. The slight decrease in the gap mode’s field strength for the bacteriophage-coated Au NP sample was caused by the surrounding environment index change.

### 3.2. Dynamic Response of M13 Bacteriophage

**Figure 3:**
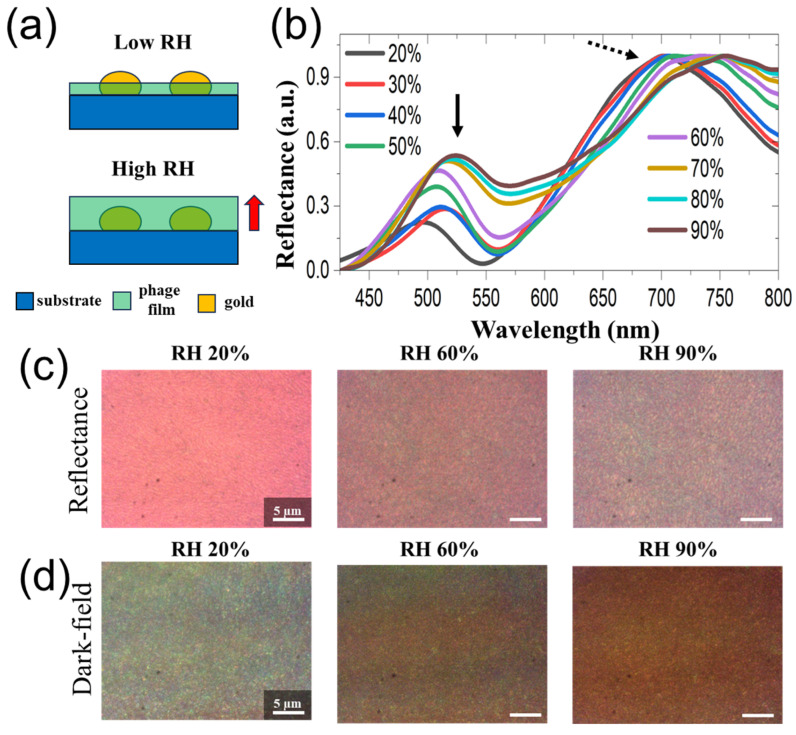
(**a**,**b**) The experimental reflectance spectra as a function of increased humidity levels varied from 20 to 90% on M13 bacteriophage/Au NPs/SiO_2_/Si samples. The concentration of M13 bacteriophage was chosen to be 10 mg/mL. Recorded CCD color images were used for reflectance (**c**) and dark field (**d**) at longer resonance wavelength positions to gain increased humidity levels. The scale bar is 5 µm for captured CCD images.3.3. Understanding the Optical and Geometrical Properties as a Function of Dynamic Response.

Utilizing our home-built chamber, we varied humidity levels to study the dynamic response of M13 bacteriophage biomaterial and its influence on the optical properties of plasmonic nanoparticles. We had chosen the plasmonic NP–biomaterial thin film, which was spin-coated with a 10 mg/mL M13 bacteriophage concentration. We chose this M13 bacteriophage’s concentration-based thickness for two reasons: it had a better dynamic response with a thicker layer and, simultaneously, a longer wavelength resonance position. The thinner M13 bacteriophage layer tends to have minimal dynamic response upon humidity exposure. The humidity levels (relative humidity percentage or RH %) ranging from 20 to 90% were applied into the chamber, and reflectivity spectra from the samples were measured. Figure 3a displays the dynamically responded reflectivity spectra with two significant optical properties. Firstly, at a shorter wavelength of around ~500 nm, a minor or negligible resonance peak position shift was observed for varied RH % levels (solid black arrow). Secondly, a clear redshift at longer wavelength resonance positions was significantly observed (dotted black arrow). The solid black arrow region with negligible changes in resonance wavelength position(s) as a function of varied RH % levels originated from the NP mode. A dominant red-shifting optical characteristic observed at the dotted black arrow region originated from the gap mode (better gap–plasmonic coupling and, hence, brighter optical mode). We also simultaneously recorded reflectivity and dark-field images for the corresponding changes in the sample upon varying RH % levels (Figure 2b,c). A clear shift in captured CCD images revealed optical color changes when the humidity levels were increased. We note that the above results can be reproduced multiple times, displaying a critical advantage in employing a M13 bacteriophage biomaterial in these nanostructures.

**Figure 4:**
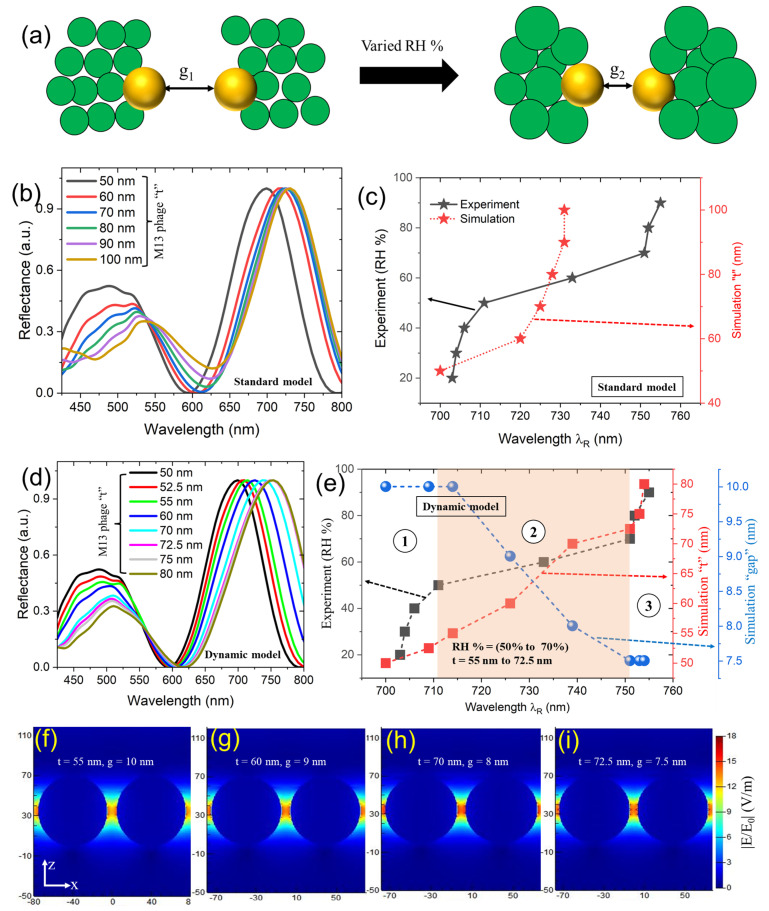
(**a**) Understanding the optical and geometrical properties of the sample via standard and dynamic simulation models. We use interparticle gap distances termed “g_1_” at 20% humidity level and “g_2_” at 90% humidity level. For a standard model, g_1_ = g_2_; for a dynamic model, g_1_ > g_2_. The green colored circles represent the schematic cross-section of the M13 bacteriophage. The simulated results for a standard model design (**b**,**c**) and a dynamic model design (**d**,**e**). The shaded orange color region in (**e**) is used to distinguish the regions indicated from 1–3. (**f**–**i**) Cross-sectional XZ electric field amplitude profiles taken for plasmonic nanostructure designs with M13 bacteriophage thicknesses ranging from 55 to 72.5 nm that display gap mode-based optical properties. The “x” and “z” axes numbers are measured in nanometers.

To understand the dynamic response-based properties of our sample in detail, we carried out 3D FDTD simulations to interpret geometrical and optical properties. Utilizing a simulated model, as seen in Figure 2h, did not work well here. In the standard model, the g_1_ = g_2_ condition was assigned to have increased M13 bacteriophage thickness (Figure 4a). The simulated (labeled as a standard model) reflectance spectra (NP diameter of 70 nm, gap = 10 nm) as a function of varied M13 bacteriophage thickness ranging from 50 nm to 100 nm (10 nm steps) studies reveal that red shifting of longer wavelength resonances occurred (λ_R_ = 731 nm) until M13 bacteriophage thickness “t” of 90 nm and became approximately consistent after that (Figure 4b). In case of experimental reflectance, the maximum red shift obtained in resonance wavelength position before becoming consistent was around ~λ_R_ = 751 nm (Figure 4c). Here, we introduce a dynamic model approach: by applying increased RH % levels, we model the structure based on g_1_ > g_2_. We assume that the decrease in g_2_ happens due to the swelling behavior of M13 bacteriophage biomaterial upon its exposure to variable humidity conditions [7]. As the M13 bacteriophage’s geometry swells in three dimensions, it induces the movement or change in NPs positions. Additionally, the water droplets formed on M13 bacteriophage structures induce a change in the effective index, which, in turn, can contribute to the gap plasmonic properties. This outcome leads to a decrease in interparticle distance, or g_2_, being noted compared to g_1_ (g_2_ < g_1_).

Figure 4d,e shows the simulated reflectance data based on our dynamic model design, which agrees with an experimental trend. To discuss it in detail, we plotted point type data extracted from the simulated and experimental results that were taken at a longer wavelength resonance (λ_R_), relating experimental RH % to simulated M13 bacteriophage thickness “t” and gap data (Figure 4e). We divided the resonance wavelength (λ_R_) regions into three parts. In part one (1), for RH % levels between 20% and <50%, the red shifting of λ_R_ was tunable at a small range (703–711 nm), and the trend very much agrees with the simulations (700–714 nm). In this situation, the predicted M13 bacteriophage thickness range varies from 50 to 55 nm, and the gap size remains unchanged (10 nm). The significant changes started in part two (2): from RH % of 50 to 70%, a drastic red shift in experimental λ_R_ was noted (711 nm to 751 nm). By correlating the data obtained via the dynamic model design at part two (2), M13 bacteriophage thickness “t” varied between >55 and 72.5 nm, with λ_R_ shifting from >714 to 751 nm. The most exciting part is the change in the interparticle distance, or gap size, from 9 to 7.5 nm. In part three (3), RH % levels changed from 70 to 90%, and similar λ_R_ peak position changes were recorded (with negligible differences). The simulated results in the third part (3) displayed a similar λ_R_ response (unchanged gap size of 7.5 nm and M13 bacteriophage thickness “t” in the range >72.5 to 80 nm). The position of the M13 bacteriophage layer plays a crucial role in inducing a shift in the peak resonance wavelength of plasmonic NPs. Specifically, when the bacteriophage layer is deposited on top of the NPs, a significant red shift in peak resonance wavelength position can be achieved. Conversely, when the bacteriophage layer is deposited below the metallic NPs, a blue shift from the original peak resonance wavelength position can occur due to the increased thickness of the dielectric layer. As both of these roles can be achieved based on how we position the M13 bacteriophage layer, it is possible to realize various plasmonic applications.

Two kinds of scenarios could be interpreted from these three parts. Firstly, in parts one and three, a minimal redshift in λ_R_ peak positions, along with unchanged gap sizes, was noted. Secondly, a drastic red shift in λ_R_ peak positions happened, which was induced by the combination of reduced gap distances between NPs and increased M13 bacteriophage thickness “t”. The swelling of the M13 bacteriophage caused the decreased gap distances between NPs as a function of increased RH % levels. Part two (2) displayed significant importance in terms of utilizing the M13 bacteriophage biomaterial as a dielectric layer film on top of the plasmonics structure for optical applications. The advantage of dynamically tunable M13 bacteriophage biomaterial can be illustrated by comparing Figure 4c,e. When employing commonly used dielectric layer materials, like SiO_2_, limits in obtaining maximum longer wavelength resonances can be visibly seen (Figure 4c) as a function of increasing thickness. But, in the case of dynamically tunable M13 bacteriophage biomaterial, with a relatively small thickness, λ_R_ red shifted further (Figure 4e) compared to Figure 4c data. This result is critically important in fabrication and becomes advantageous in terms of demonstrating multiwavelength studies from a single sample by utilizing this biomaterial thin film. For example, to name a few, such potential applications can involve nano-particle-on-mirror (NPOM) designs, sensors, meta-optics, etc.

Plasmonic nanostructures offer us numerous opportunities to develop efficient optical designs based on near-field enhancement strength, optical modes, and localized surface plasmon resonance (LSPR) properties, which empower various applications [34,47,48,49]. Combining these attractive plasmonic features with biomaterial helps us to realize highly selective and sensitive sensing platforms. As biomaterial-based plasmonic nanostructures support humidity-based optical changes, this result opens up multi-wavelength applications from a single design, rather than from the fabrication of multiple nanostructure designs. From our previous work (M13 bacteriophage deposited first and plasmonic NPs spin-coated on top), we reported how the non-uniform thickness of M13 bacteriophage influences the LSPR properties [34]. This work (plasmonic NPs spin-coated first and M13 bacteriophage deposited on top of it) helped us to realize that the humidity-based changes played a crucial role in switching optical properties based on NP positioning, gap size “g” changes, and effective index modification caused by the presence of moisture upon the introduction of varying humidity levels. We believe that this type of study will help to provide essential insights when designing integrated plasmonic–biomaterial sensor nanostructures.

In particular, the genetic engineering of M13 bacteriophage introduces thousands of surface protein modifications that can be utilized for highly sensitive and selective bio-sensing applications in point-of-care diagnostics, agriculture, and environmental protection [11,14,15,18,19,26,27]. In the case of NPOMs, with a single-bottom-faceted NPOM design that can exhibit superior optical characteristics, even in cases of larger spacer gap sizes, the genetically engineering M13 bacteriophage can open various plasmonic studies and applications alongside the dynamic geometry change. We note that the dynamic changes caused by the M13 bacteriophage biomaterial are reversible, and samples can be restored to their original characteristics on a longer lifetime basis.

## 4. Conclusions

In this study, we conducted experimental investigations centered on a plasmonic nanoparticle (NP)–biomaterial thin film structure, specifically incorporating the M13 bacteriophage. We demonstrated its ability to manipulate the thickness of the M13 bacteriophage layer by modulating either the bacteriophage concentration or humidity conditions. The observed optical characteristics from this study were comprehensively analyzed via two simulation models: a standard model and a dynamic model. The dynamic simulation model offered in-depth predictions and analyses of both the optical and geometrical attributes, including the dynamic response of M13 bacteriophage thickness and the subsequent alteration in interparticle spacing between NPs. The model successfully interpreted the humidity-induced alterations observed in the experiment. Furthermore, we demonstrated that plasmonic nanostructures can be rendered reactive by applying a coating of M13 bacteriophage. Such functionalized nanostructures could potentially be harnessed in sensor applications. Beyond these findings, the successful fabrication, reproducibility, and reversibility, along with the capability to genetically engineer the surface proteins of the M13 bacteriophage biomaterial, suggest a broad range of prospective applications. These applications include, but are not limited to, optics, sensors, plasmonics, and surface-enhanced Raman spectroscopy. The possibility of achieving precise selectivity and high response rates adds to the potential impact in these fields.

## Data Availability

Not applicable.

## Supplementary Materials

The following supporting information can be downloaded at: https://www.mdpi.com/article/10.3390/s23136011/s1, Figure S1: Schematic illustration of simulated model involving M13 phage/Au NPs/SiO2/Si nanostructure and its geometrical parameters. Depiction of a cross-sectional views (**a**,**b**) and top view (**c**). A plane wave source is used to excite the sample in a normal direction from the top (+Z direction) with an incident electric field of E_0_. The NP diameter D = 70 nm, interparticle or gap distance termed as “g”, and thickness of M13 phage as “t”. The period is set to “D+g”. We used 5*period condition in X and Y directions (periodic boundaries—red solid line as shown in **c**). PML boundary condition applied in Z direction. Figure S2. Simulated reflectance spectra from a single (**a**) and dimer (**b**) NP model (M13 phage/Au NPs/SiO_2_/Si). The black solid line spectra represent the nanostructure without M13 phage. In both these cases, a PML boundary conditions were applied in XYZ directions. Figure S3. Measure height profiles for the samples coated with 6 mg/mL (**a**), 8 mg/mL (**b**), and 10 mg/mL (**c**) M13 phage concentration. The solid dotted lines is used for easy illustration of an average height obtained from the samples with different M13 phage’s concentration (30 nm, 40 nm, and 50 nm in order, respectively). Figure S4. Phage film thickness as a function of humidity as measured by atomic force microscope (AFM). The AFM measurements was conducted to observe the noticeable swelling of the M13 layer. (**a**) Height profiles of M13 phage film depending on various humidity measured by AFM. (**b**) Three-dimensional AFM images with 20% humidity. (**c**) Three-dimensional AFM images with 90% humidity.
