# Supplementary material for: Understanding the Role of M13 Bacteriophage Thin Films on a Metallic Nanostructure through a Standard and Dynamic Model
Source: Sensors (Basel). 2023 Jun 28;23(13):6011. doi: 10.3390/s23136011 (PMC10347125; doi:10.3390/s23136011)
Supplement: Supplementary file 1 [file sensors-23-06011-s001.zip › sensors-2442480-supplementary.pdf]

Supporting Information

Understanding the Role of M13 Bacteriophage Thin Films on a Metallic Nanostructure through a Standard and Dynamic Model

Thanh Mien Nguyen [1,†], Cheol Woong Choi [2,3,†], Ji-Eun Lee [3,4,†], Damun Heo [5], Ye-Won Lee [5], Sun-Hwa Gu [5], Eun Jeong Choi [1], Jong-Min Lee [5,6,*], Vasanthan Devaraj [1,*] and Jin-Woo Oh [1,7,*]

1 Bio-IT Fusion Technology Research Institute, Pusan National University, Busan 46241, Republic of Korea
2 Department of Internal Medicine, Medical Research Institute and Research Institute for Convergence of Biomedical Science and Technology, Pusan National University Yangsan Hospital, Yangsan-si 50612, Republic of Korea
3 School of Medicine, Pusan National University, Yangsan 50612, Republic of Korea
4 Department of Ophthalmology, Research Institute for Convergence of Biomedical Science and Technology, Pusan National University Yangsan Hospital, Yangsan 50612, Republic of Korea
5 School of Nano Convergence Technology, Hallym University, Chuncheon 24252, Republic of Korea; yw330@naver.com (Y.-W.L.); 20203301@hallym.ac.kr (S.-H.G.)
6 Center of Nano Convergence Technology, Hallym University, Chuncheon 24252, Republic of Korea
7 Department of Nanoenergy Engineering and Research Center for Energy Convergence Technology, Pusan National University, Busan 46214, Republic of Korea
* Correspondence: jmlee@hallym.ac.kr (J.-M.L.), devarajvasanthan@gmail.com (V.D.); ojw@pusan.ac.kr (J.-W.O.)
† These authors contributed equally to this work.

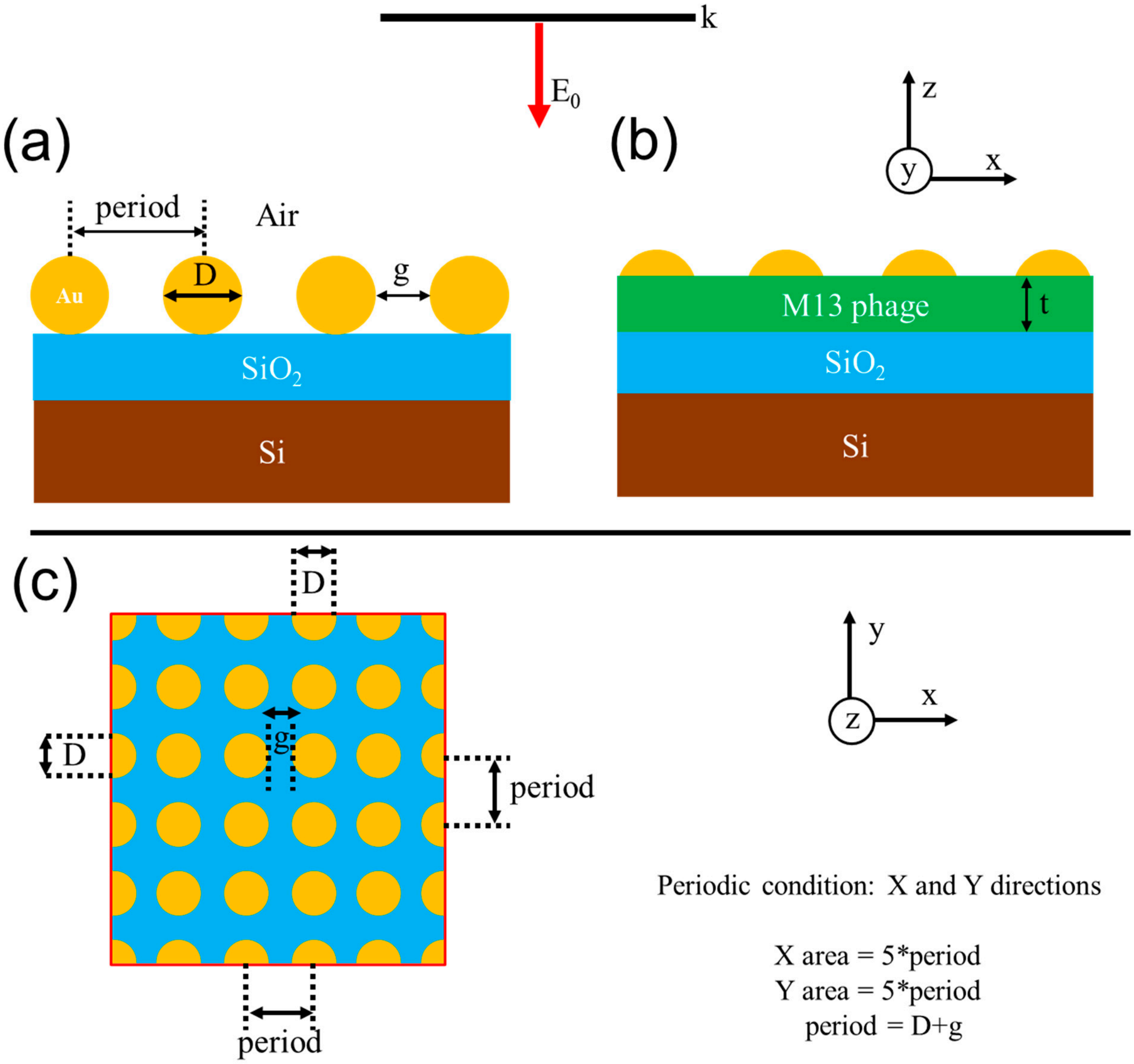

Figure S1. Schematic illustration of simulated model involving M13 phage/Au NPs/SiO_2/Si nanostructure and its geometrical parameters. Depiction of a cross-sectional views (**a** – **b**) and top view (**c**). A plane wave source is used to excite the sample in a normal direction from the top (+Z direction) with an incident electric field of E_0. The NP diameter D = 70 nm, interparticle or gap distance termed as "g", and thickness of M13 phage as "t". The period is set to "D+g". We used 5*period condition in X and Y directions (periodic boundaries – red solid line as shown in **c**). PML boundary condition applied in Z direction.

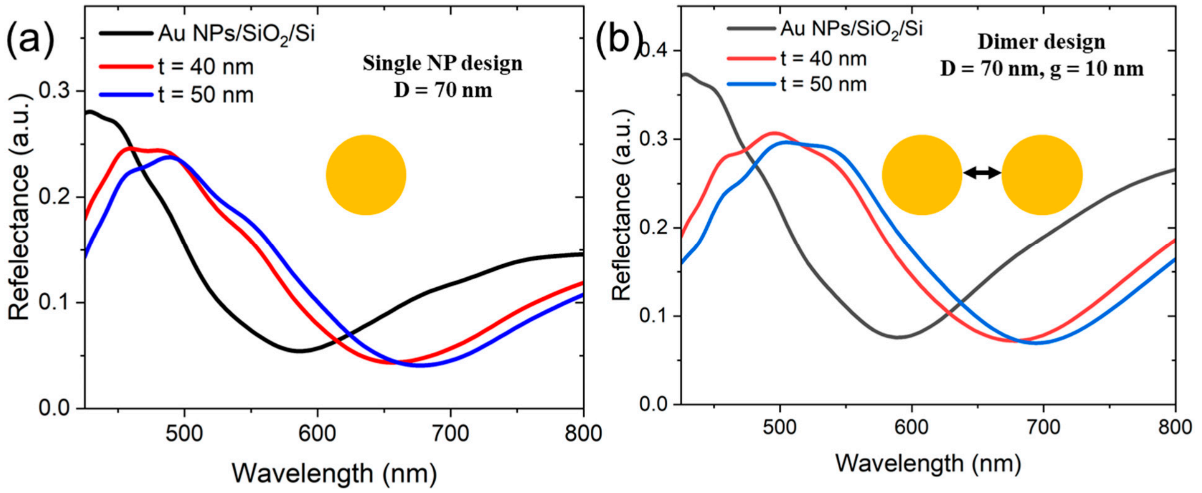

Figure S2. Simulated reflectance spectra from a single (a) and dimer (b) NP model (M13 phage/Au NPs/SiO_2/Si). The black solid line spectra represent the nanostructure without M13 phage. In both these cases, a PML boundary conditions were applied in XYZ directions.

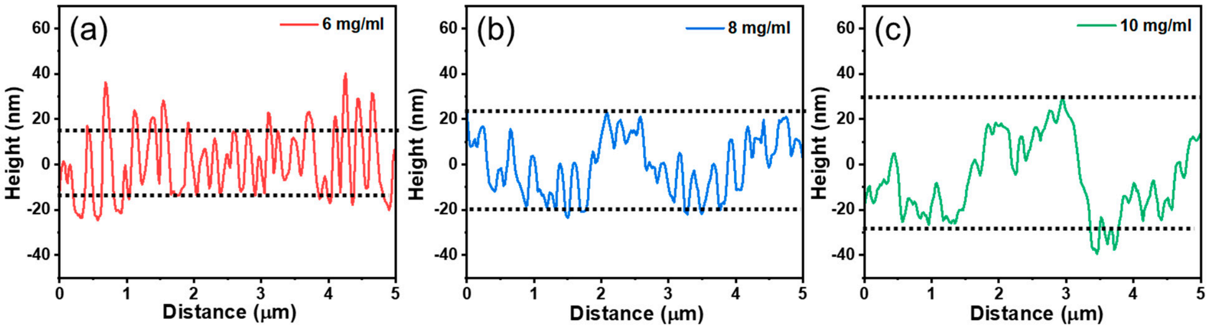

Figure S3. Measure height profiles for the samples coated with 6 mg/ml (a), 8 mg/ml (b), and 10 mg/ml (c) M13 phage concentration. The solid dotted lines is used for easy illustration of an average height obtained from the samples with different M13 phage's concentration (30 nm, 40 nm, and 50 nm in order, respectively).

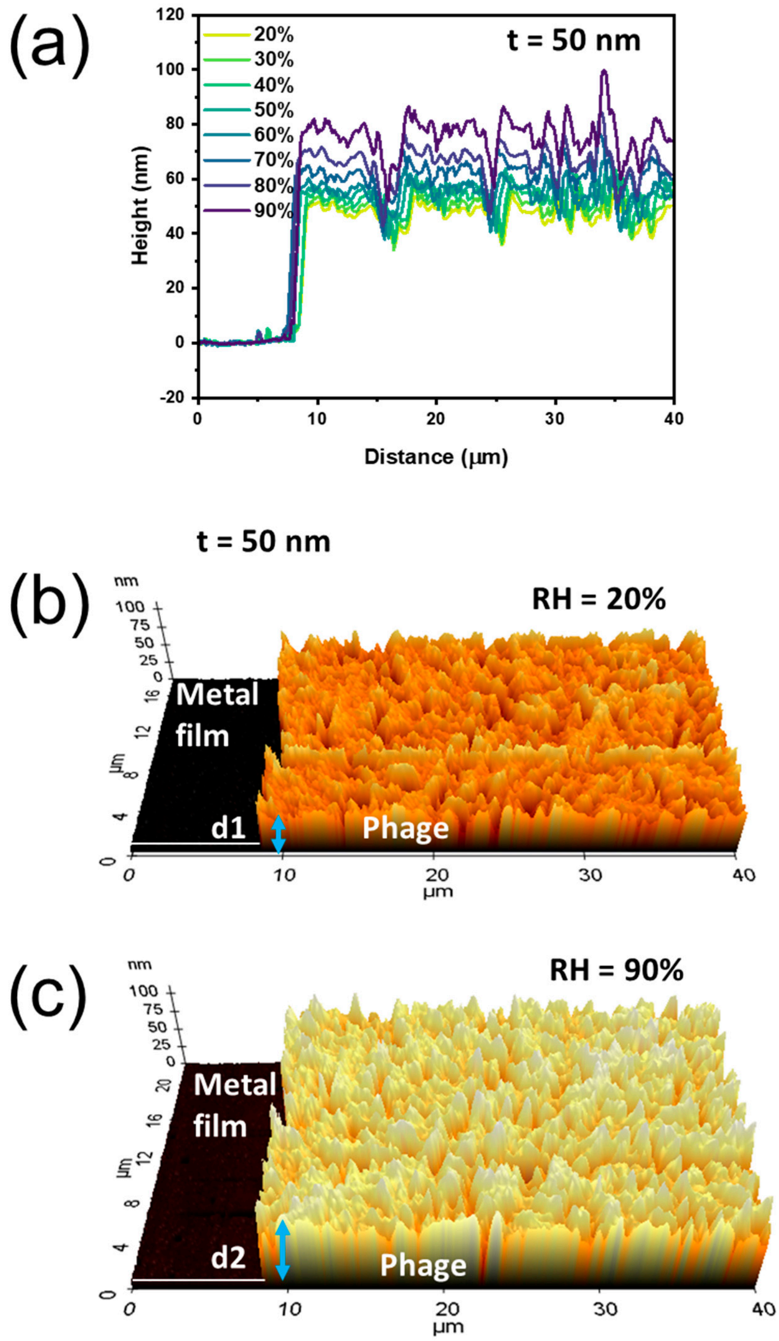

Figure S4. Phage film thickness as a function of humidity as measured by atomic force microscope (AFM). The AFM measurements was conducted to observe the noticeable swelling of the M13 layer. (a) Height profiles of M13 phage film depending on various humidity measured by AFM. (b) Three-dimensional AFM images with 20% humidity. (c) Three-dimensional AFM images with 90% humidity.
